# Temporal dynamics of *Grapevine red blotch virus* accumulation in grapevine leaves is influenced by fruit maturity stages

**DOI:** 10.1007/s00705-026-06634-0

**Published:** 2026-05-20

**Authors:** Prem P. Singh, Kishorekumar Reddy, Hazel Scully, Arpa M. Boghozian, Cristina Medina-Plaza, Anita Oberholster, Mysore R. Sudarshana

**Affiliations:** 1 Department of Viticulture & Enology, University of California, Davis, CA USA; 2 USDA-ARS, Department of Plant Pathology, University of California, Davis, CA USA

## Abstract

Grapevine red blotch disease (GRBD) poses a serious threat to viticulture in the United States. Grapevine red blotch virus (GRBV), a member species of *Grablovirus vitis* (family *Geminiviridae*), the causal agent of GRBD, disrupts grapevine physiology and metabolism, thereby interfering with the natural processes of fruit ripening. To date, there is limited knowledge about how grape maturity stages influence the timing of changes in viral load and disease progression. This study elucidates the effect of fruit maturity stages on GRBV accumulation in *Vitis vinifera* L. cv. Merlot grapevines in a Central Coast vineyard in California. Petioles from six basal leaves at random from previously GRBV-infected vines were collected at pre-véraison, véraison, post-véraison, and harvest stages, across two years (2021 and 2022). The viral copy number was quantified using digital PCR. The study identified significant differences (*p* < 0.05) in GRBV copy number across different maturity stages in 2021, irrespective of the year of infection. The lowest viral load was observed during the pre-véraison stage, and the highest at harvest, indicating progressive viral accumulation as the grapevines development progressed. In 2022, however, no significant difference in viral load across maturity stages was detected, an outcome attributed to extreme heat spikes coinciding with sampling periods. Our findings highlight the interplay between GRBV accumulation, grape development, and environmental parameters.

**Supplementary Information:**

The online version contains supplementary material available at 10.1007/s00705-026-06634-0.

## Introduction

The viniculture industry, contributing ~$276 billion to the US economy in 2022, faces a significant threat from grapevine red blotch disease (GRBD) [1]. Because of its adverse effects on wine quality, GRBD is a major concern for the U.S. wine industry, especially for premium wine production [2–4]. While grapevine red blotch virus, (GRBV) (*Grablovirus vitis*, family *Geminiviridae*), is present in vineyards and/or germplasm collections of countries such as Argentina [5], Australia [6], Canada [7], Italy [8], India [9], Mexico [10], and South Korea [11], the impact of GRBD is of huge concern in California [4] which accounts for over 80% of the U.S. wine produced. The discovery of the alfalfa treehopper, *Spissistilus festinus* (family Membracidae), capable of spreading GRBV in vineyards, has further raised major concerns for the wine industry [12, 13].

Symptoms of GRBV infection vary by cultivar: red-berried cultivars show red blotches on leaves, while white-berried cultivars exhibit mild to moderate chlorosis, occasionally with interveinal necrosis. GRBD negatively affects wine quality, resulting in economic losses of $2,200 to $68,600 per hectare in the United States [14]. The virus disrupts grapevine physiology, interfering with fruit ripening [15]. This leads to a decrease in sugar and anthocyanin accumulation, both crucial for wine quality. Wines made from the infected grapevines experience lower alcohol content, reduced color intensity, and altered sensory profiles, making them less competitive in the market [3]. Most of the widely grown wine grape varietals, including Cabernet Sauvignon, Chardonnay, Merlot, and Pinot Noir, are known to be affected.

To date, there is limited understanding of how grape maturity stages influence the temporal dynamics of viral load, which is considered a key parameter in efficient virus transmission and symptom expression [16]. Previous studies have focused on the seasonal pattern of virus copy number primarily in the early and late growing seasons, establishing the relationship between virus copy number and transmission by the insect vector, *S. festinus* [17]. However, these studies did not explore the temporal dynamics of viral load progression across grape maturity stages. Also, there is no study on whether the virus titer changes in the years following new infections using extremely sensitive detection techniques. In recent years, digital PCR (dPCR) has gained popularity as an advanced diagnostic tool. Unlike traditional PCR and quantitative PCR (qPCR), which rely on relative quantification and standard curves to estimate copy numbers, dPCR delivers absolute quantification by partitioning each reaction into thousands of partitions, enabling precise counting of viral genome copies. This level of accuracy is vital for studies analyzing temporal dynamics across multiple seasons and years, as relative methods are limited when comparing viral loads from samples collected under varying conditions or at different times. Fluctuations in amplification efficiency or reference gene expression can complicate such comparisons. Conversely, dPCR provides absolute copy numbers that are directly comparable across seasons, infection groups, and developmental stages, which align well with the mixed-effects modeling approach used in this study. Additionally, dPCR’s high sensitivity and precision make it particularly effective for detecting low viral loads [12, 18], especially for early detection and accurate quantification of GRBV in grapevine tissues [12].

In the present study, we attempted to determine the temporal dynamics of GRBV in Merlot grapevines in a California vineyard using dPCR to measure viral loads at different grape-maturity stages and several years post-infection. A key goal is to identify maturity stages with the highest viral accumulation to determine the optimal timing for field-deployable diagnostic tests.

## Materials and methods

### Site selection

A vineyard located at Paso Robles (Central Coast, California, USA), planted in 2009 with *Vitis vinifera* L. cv. Merlot clone 12 grapevines grafted on 1103P rootstock were selected for this study. The vineyard followed standard regional management practices. In 2016, the grapevines were tested for GRBV, grapevine leafroll-associated viruses-1 (GLRaV-1), GLRaV-3, and GLRaV-4, and grapevine rupestris stem pitting-associated virus by Agri-Analysis LLC (www.agri-analysis.com). Grapevines testing positive for the tested viruses were eliminated from the study and from 2017 onwards, the grapevines were tested only for GRBV by qPCR.

### Sample collection

Symptomatic grapevines that tested positive for GRBV (RB+) for the first time in 2016, 2019, 2020, 2021, and 2022 were selected for the study. Each year, a minimum of 50 asymptomatic grapevines were tested for GRBV infection, and the newly infected grapevines were identified for sampling in 2021 and 2022.

These vines were divided into five groups based on the year of infection (2016, 2019, 2020, 2021, and 2022). For each group, eight vines were selected as biological replicates for both RB(−) and RB(+) conditions. For 2022, only four replicates were used as there were not enough newly infected grapevines. Sampling and viral copy number analysis were conducted over the growing seasons of 2021 and 2022. Six basal leaves from randomly chosen shoots from grapevines each year at four maturity stages- pre-véraison (green berries), véraison (50% of berries showing color and softening), post-véraison (100% of berries colored and soft), and harvest (fully mature berries with TSS 25°Brix) for virus copy number analysis.

### Nucleic acid extraction and testing for grapevine red blotch virus (GRBV) by qPCR

Unless otherwise mentioned, all equipment and reagents used for qPCR and dPCR were from ThermoFisher Scientific Inc, Waltham, MA, USA. Total nucleic acids (TNA) were extracted from grapevine petiole tissues (150 mg) using MagMAX™-96 Viral RNA Isolation Kit on a KingFisher Flex DNA/RNA purification instrument. Nucleic acid fraction bound on the beads was eluted in 100 µl of nuclease free water.

Quantitative PCR (qPCR) was conducted to detect GRBV in 20 µl reactions containing 1 µl of TNA extract, 0.15 µM each of primers GRBaV-F1580 and GRBaV-1693R [12], 2% polyvinylpyrrolidone-40, 0.3 µl of Stratagene Reference Dye (1:500), 10 µl of SsoFast EvaGreen Supermix (Bio-Rad Inc., Hercules, CA), and nuclease-free water to make up the remaining volume. The qPCR was conducted on a QuantStudio 6 Flex Real-Time PCR System with an initial denaturation at 95 °C for 2 min, followed by 35 cycles of 95 °C for 30 s, 62 °C for 1 min, and 72 °C for 1.5 min. High-resolution melting (HRM) analysis was performed post-amplification to verify the identity of the amplified products. The HRM conditions included 95 °C for 1 min, 55 °C for 30 s, and 95 °C for 30 s, with continuous fluorescence measurements. Samples were considered positive if they produced a measurable quantification cycle (Cq) value < 35 and the amplicon exhibited a melting temperature consistent with that of a positive control.

### TNA quality assessment and GRBV copy number quantification

Prior to GRBV quantification by dPCR, the quality and integrity of the TNA extract were assessed by qPCR targeting the endogenous *V. vinifera* actin gene. The assay included TaqMan probe (5’-FAM-CAC TGT GCC AAT TTA TGA AGG TTA TGC ACT TC-MGB-NFQ-3’), along with the forward primer 5’- GTA TTG TGC TGG ATT CTG GTG AT-3’ and reverse primer 5’- GCA AGG TCA AGA CGA AGG ATA G-3’ [19]. Reactions were assembled in 20 µl volumes containing 10 µl of TaqMan™ Fast Advanced Master Mix (2X; Applied Biosystems, USA), 1 µl of actin TaqMan assay reagents [19], 7 µl of nuclease-free water, and 2 µl of TNA extract. Thermal cycling was performed on a QuantStudio 6 Flex Real-Time PCR System (ThermoFisher Scientific Inc., Waltham, MA) using the following protocol: 50 °C for 2 min, 95 °C for 10 min, followed by 40 cycles of 95 °C for 15 s and 60 °C for 1 min [19]. A quantification cycle (Cq) value below 35 was used to confirm the presence of intact amplifiable nucleic acids, thereby validating the sample for subsequent GRBV quantification.

Digital PCR assays for virus copy number determination were conducted using a QuantStudio 3D Digital PCR System, following the manufacturer’s instructions. Each reaction consisted of 6 µl of 1:1000 diluted TNA and 9.5 µl of reaction mixture. The latter included QuantStudio 3D master mix and custom-ordered GRBV TaqMan assay reagents. The TaqMan probe used was 5’-FAM-AGA ACT GAA GTT GAA GAA TT-MGB-NFQ-3’, along with the forward primer 5’-AAG AAT TGC ATT GAC TGA ACC TGA-3’ and reverse primer 5’-CCT AGC TCC AGG TCC AGA CG-3’ [12].

The prepared reaction mix was loaded onto a QuantStudio 3D Digital PCR Chip using a Chip Loader and run on a ProFlex Base PCR System. Thermal cycling included an initial denaturation at 95 °C for 2 min, followed by 35 cycles of 95 °C for 30 s, 65 °C for 30 s, and 72 °C for 30 s, with a final extension at 72 °C for 5 min. The chips were scanned for fluorescence data and analyzed using QuantStudio 3D Analysis Suite Cloud Software (https://apps.thermofisher.com/quantstudio3d).

### Mixed-effects model to predict the effects of maturity stages and years since infection

A linear-mixed model was employed to evaluate the effects of maturity stages and years on virus copy numbers. For the model, log_10_-transformed virus copy number was used as the response, while ‘maturity stage’ and ‘year’ were used as fixed effects (formula: log_10_(Virus Load) ~ Maturity stages + Year). The model was fit using restricted maximum likelihood (REML) to ensure unbiased estimates of variance components [20]. The primary predictors were maturity stages, representing fruit development stages, pre-véraison, véraison, post-véraison, and harvest, and the year when the virus infection of the grapevine was first noticed (2016, 2019, 2020, and 2021), accounting for temporal variability across the seasons.

Model diagnostics were performed to validate assumptions. Normality of residuals was checked using quantile-quantile (Q-Q) plots, while homogeneity of variance was inspected by plotting residuals against fitted values, and no data point was excluded as an outlier. Performance metrics for model comparison were computed using the performance package in R [20, 21].

To evaluate the effects of sampling period and year on virus copy number, various linear models were fitted with the log-transformed viral load as the response variable. The primary predictor was the sampling period, treated as a categorical variable with pre-véraison as the reference level. The variable year was treated as both a fixed effect and a random effect, allowing viral load to vary across years. Afterwards, the models were compared using the Akaike Information Criterion (AIC).

### Statistical analysis

Statistical analyses were performed using GraphPad Prism. Additionally, the mean virus copy number was presented in interleaved bar plots with standard errors.

## Results

### Yearly variations in spatiotemporal dynamics of GRBV accumulation

**Fig. 1:**
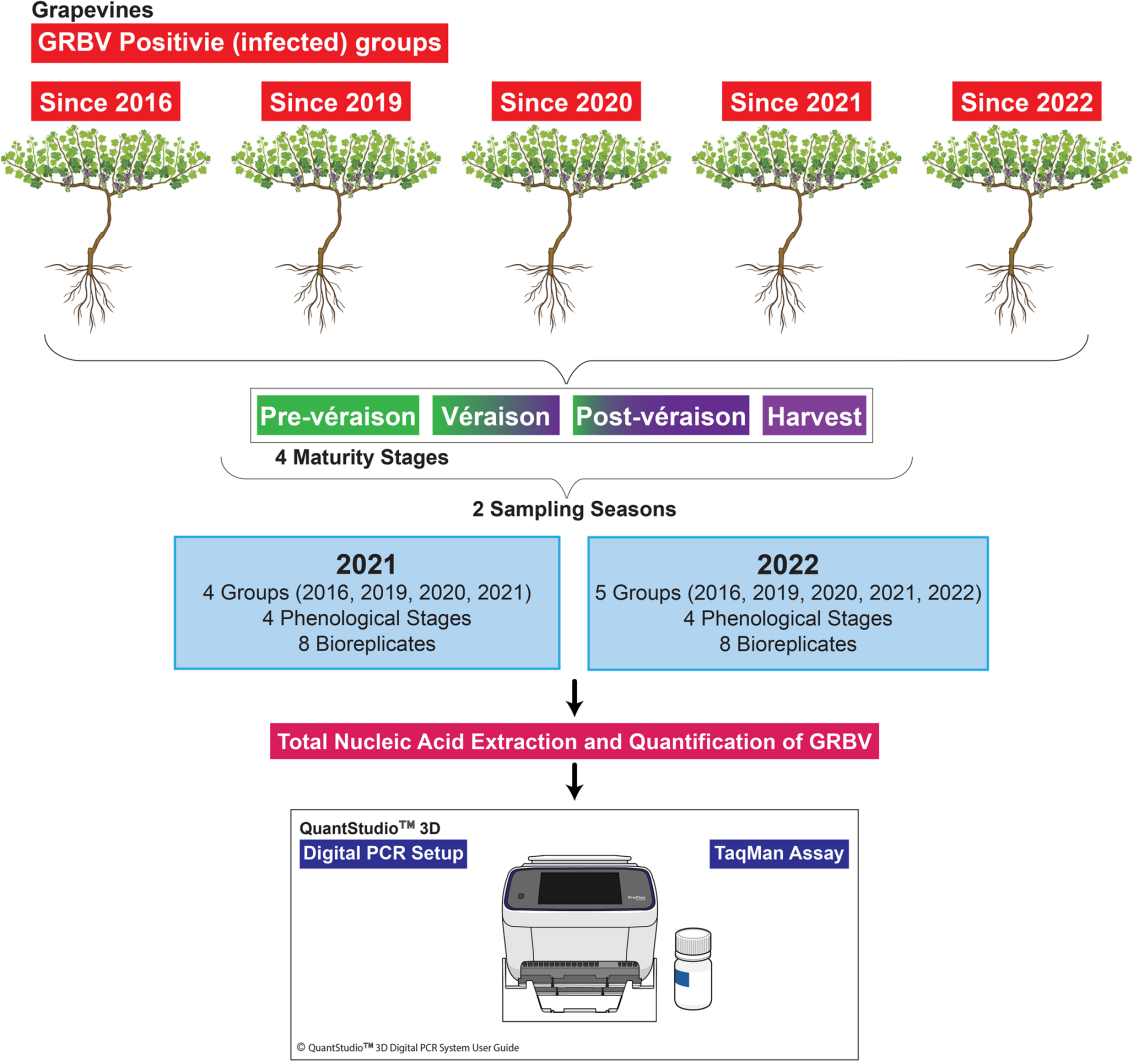
Study design and sampling overview

The present study comprised the collection of 128 samples in 2021 and 144 samples in 2022 from *V. vinifera* L. cv. Merlot grapevines, across four maturity stages: pre-véraison, véraison, post-véraison, and harvest. The vines used for the study were not infected by GRBV in the years prior to 2016 in qPCR tests. Only those found infected with GRBV for the first time in 2016, 2019, 2020, 2021, and 2022 were chosen to determine the progression of GRBV accumulation across the four fruit maturity stages in virus-infected vines over two growing seasons in 2021 and 2022 (Fig. 1).

To ensure that the nucleic acid extracts from grapevines were of sufficient quality for downstream analysis by dPCR, a subset of randomly selected samples from each season was assessed using a TaqMan qPCR assay targeting the *V. vinifera* actin gene. All tested samples had produced amplification with Quantification cycle (Cq) values below 35, confirming the integrity and suitability of the extracted TNA for downstream viral quantification (Supplementary Fig. S1). Consistent with Setiono et al. [22], use of a constitutively expressed gene such as Actin provided a reliable means to validate nucleic acid integrity and suitability for downstream molecular diagnostics.

**Fig. 2:**
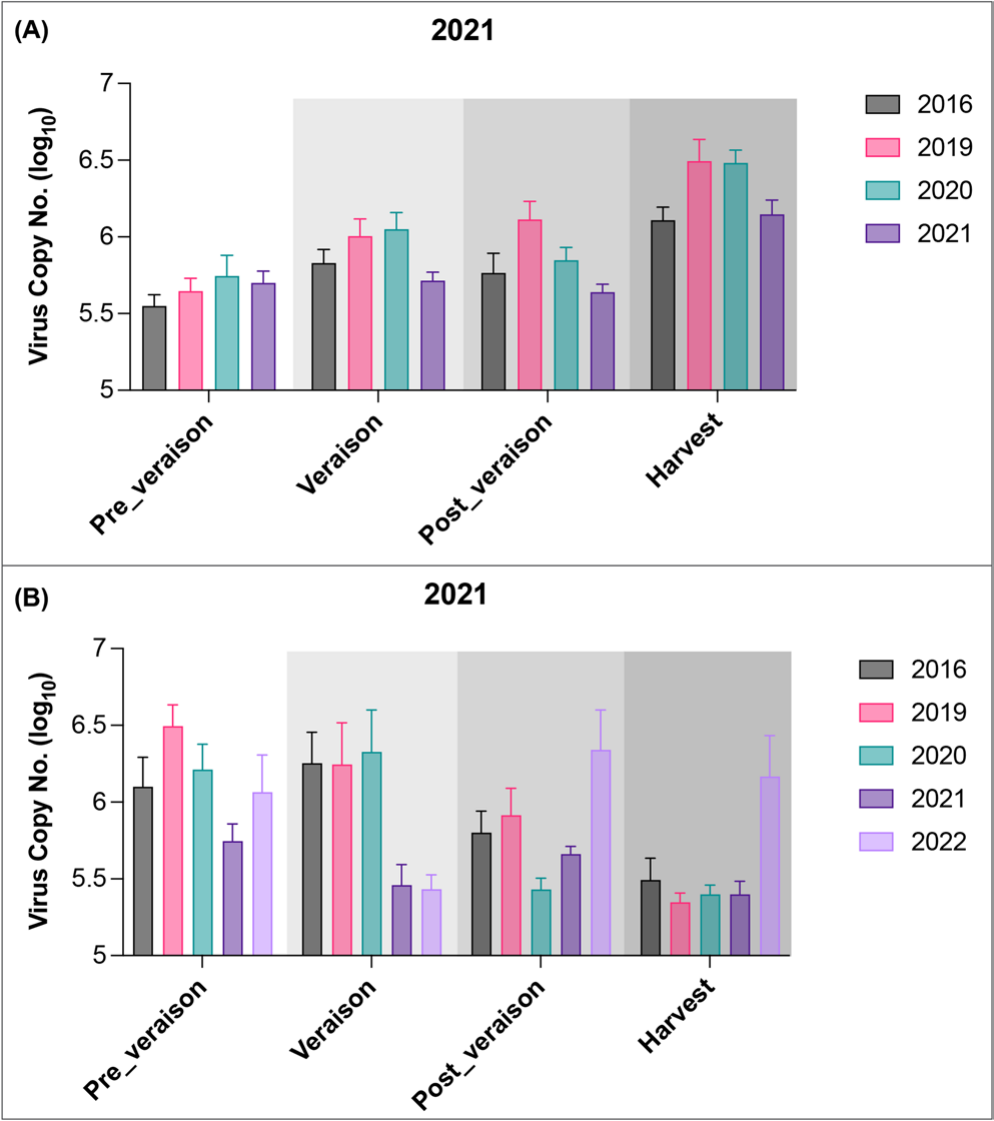
Seasonal variation in the copy number (shown on logarithmic scale) of grapevine red blotch virus across maturity stages and infection groups for sampling years (**A**) 2021 and (**B**) 2022. Solid bars represent the mean values and lines represent the standard error of the mean

There was a gradual and significant increase in the mean viral copy numbers during the 2021 season as grapevines progressed through their maturity stages (Fig. 2). The lowest viral copy number was observed at pre-véraison, with viral accumulation peaking at harvest. In contrast, samples collected in 2022 exhibited consistently lower statistically insignificant viral copy numbers, with no discernible pattern across the maturity stages or years since infection (Supplementary Fig. S2).

**Fig. 3:**
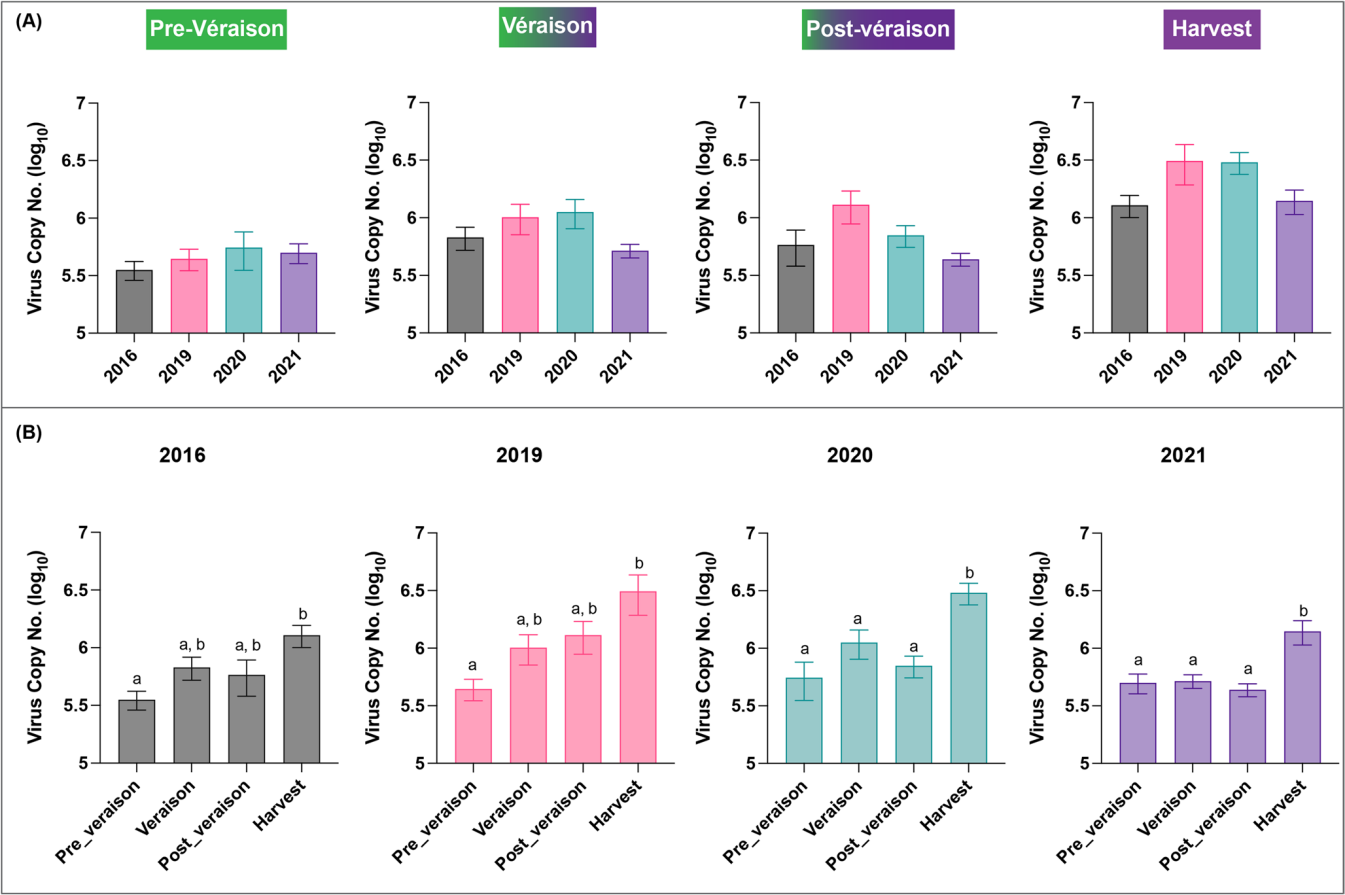
Comparative analysis of grapevine red blotch virus copy number (shown on logarithmic scale) for the 2021 growing season across (**A**) maturity stages and (**B**) different years of infection. Solid bars represent the mean values, and the lines represent the standard error of the mean

To further examine how years since infection may affect virus accumulation, virus copy numbers across different infection years (2016, 2019, 2020, and 2021) were compared at each maturity stage in Fig. 3(A). While there appeared to be some variation in virus accumulation, especially with slightly higher levels at véraison and harvest in 2019 and 2020, statistical analysis showed that these differences were not significant at any individual maturity stage. These results indicate that the number of years since the initial infection did not significantly impact GRBV copy number, indicating a relatively stable viral load pattern over time once vines were infected.

### Temporal dynamics of GRBV copy number across maturity stages

In contrast to the relatively stable viral load across years, fruit maturity stage emerged as a key determinant of GRBV accumulation (Fig. 3B). All infection years (2016, 2019, 2020, and 2021) exhibited a general increase in virus copy number from pre-véraison to harvest. However, the pattern and statistical strength of this increase varied across years.

In the 2021 growing season for grapevines infected with GRBV in 2016 and 2019, viral copy number was significantly higher (*p* < 0.01) at harvest when compared to those at pre-véraison. However, there was no significant difference between véraison and post-véraison stages (Fig. 3B). This indicates a moderate increase in viral load as the fruit matured. By contrast, for grapevines infected with GRBV in 2020 and 2021, viral accumulation was significantly higher (*p* < 0.01) at harvest compared to all earlier stages, including pre-véraison, véraison, and post-véraison, suggesting a more consistent and steep viral progression during ripening.

### Mixed effect model

To better understand the contributions of both maturity stage and year since infection on GRBV copy number, we implemented a mixed-effect modeling approach. Several model configurations were tested, incorporating ‘year’ and ‘maturity stage’ as both fixed and random effects. The optimal model, selected based on the lowest Akaike Information Criterion (AIC), included ‘maturity stage’ and ‘year’ as fixed effects, while their interaction term was excluded due to the lack of statistical significance.

The model revealed that ‘maturity stage’ had a significant impact on viral load, whereas the ‘year’ did not. Compared to pre-véraison, viral titer increased significantly at véraison (Estimate = 0.217, *p* < 0.05) and harvest (Estimate = 0.609, *p* < 0.001), while post-véraison showed a marginal increase (Estimate = 0.146, *p* ≈ 0.06), suggesting a gradual buildup of viral load as the berries matured.

Regarding the effect of the ‘year’, the model showed no significant impact on viral copy number (Estimate = 0.021, *p* > 0.05), supporting observations from Fig. 3(A) and confirming that inter-annual variation did not significantly influence GRBV accumulation. This finding highlights the dominant role of fruit maturity stages over the infection-year factor.

**Fig. 4:**
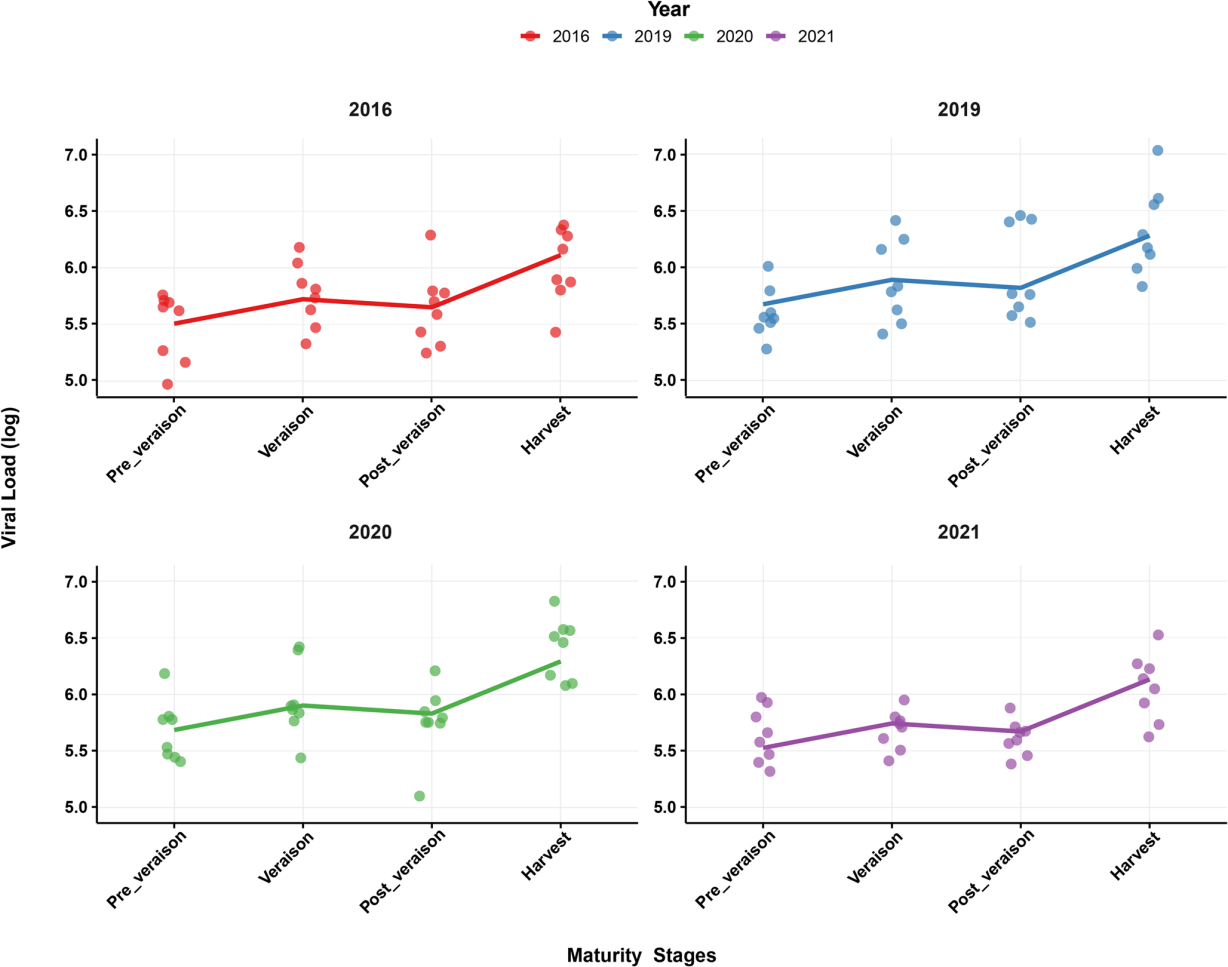
Yearly trends in fitted data of grapevine red blotch virus copy number (shown on logarithmic scale) over progressing maturity stages. Dots represent observed values for individual biological replicates, and lines show the fitted values from the linear mixed-effects model

Model diagnostics indicated that residuals were normally distributed, and homoscedasticity was maintained, affirming the validity of the model. Furthermore, estimated marginal means highlighted significant differences across maturity stages, particularly the sharp increase at the harvest stage (Fig. 4).

### Environmental influence on GRBV copy number

**Fig. 5:**
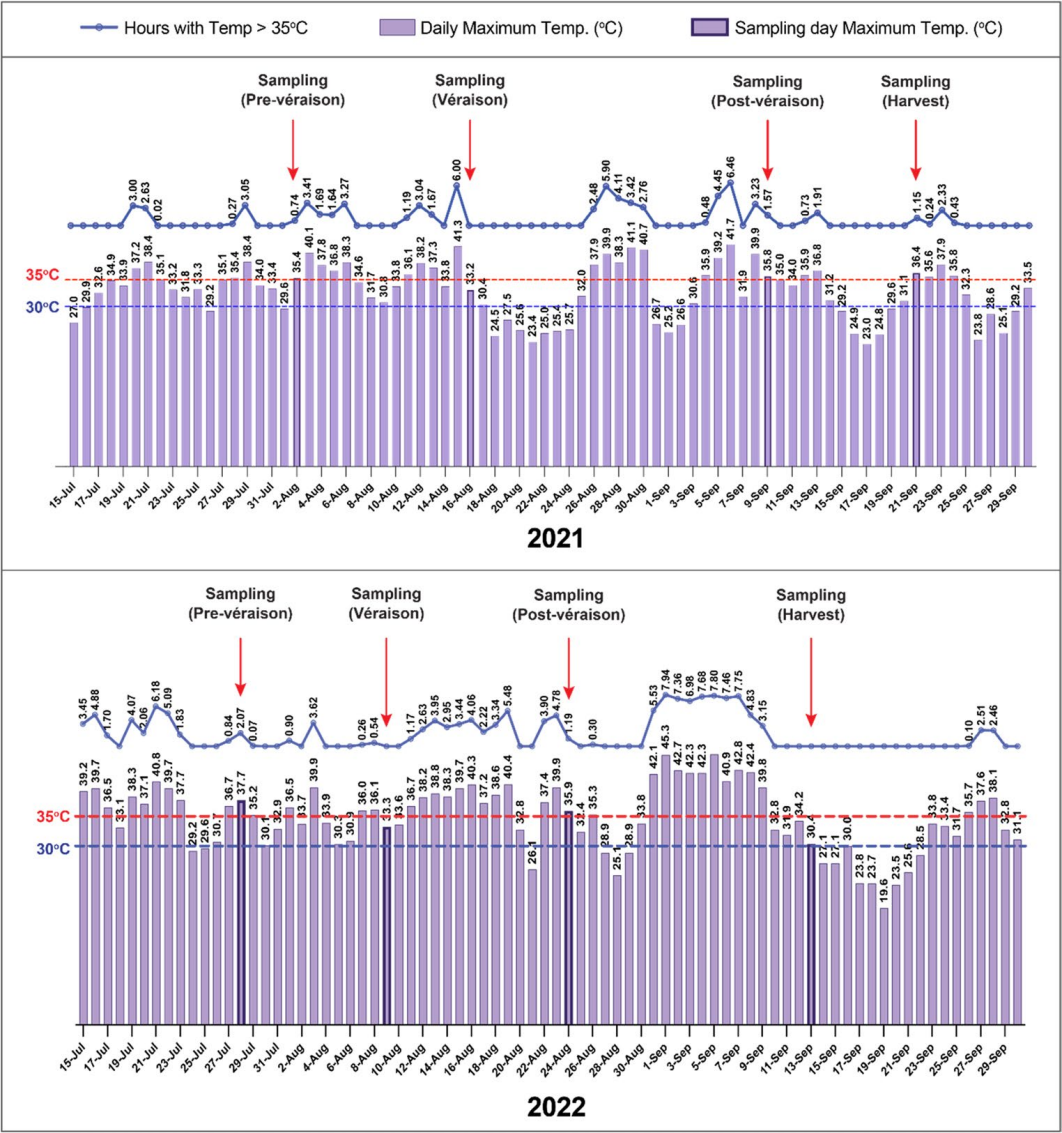
Profile of maximum temperature (°C) across sampling periods in 2021 and 2022. Bars represent daily maximum temperatures; highlighted bars indicate sampling days at each of the four maturity stages (pre-véraison, véraison, post-véraison, and harvest). The black line indicates the number of hours per day when temperatures exceed 35 °C

The differences between the 2021 and 2022 datasets highlight the significant impact of environmental factors on GRBV spatiotemporal dynamics. In 2021, viral accumulation followed a predictable increase with grapevine development, whereas in 2022, a spike in summer appeared to disrupt this pattern. This suggests that GRBV behavior and accumulation can vary widely depending on environmental conditions. Specifically, the extreme heat in 2022 significantly reduced viral load across all maturity stages, with the strongest effects observed at post-véraison and harvest (Supplementary Fig. S2). In 2022, sampling dates coincided with heat waves exceeding 35 °C for several days (Fig. 5), especially during the post-véraison and harvest stages, which aligned with the observed reduction in viral load.

## Discussion

The relationship between the GRBV load and disease progression in grapevines has been of great interest in recent years, with an emphasis on seasonal dynamics, diagnostic methods, and impacts on vine health and fruit quality. In British Columbia, Poojari et al. [7] studied the incidence, distribution, and genetic diversity of GRBV, uncovering variability in infection rates and potential links between viral diversity and symptom severity. Building on this, Kahl et al. [20] investigated seasonal dynamics and identified optimal diagnostic windows based on peak viral titer. In Southern Oregon, DeShields and Achala [16] also evaluated diagnostic efficacy and reported detection inconsistencies in symptomatic versus asymptomatic infections in Pinot noir vines. These findings, together with earlier observations from Oregon’s Willamette Valley [23], suggest that cold environmental conditions may modulate disease expression through delayed symptom onset, highlighting the potential role of regional climate in influencing GRBV progression.

Recently, Flasco et al. [17] determined the seasonal variation in GRBV titer in relation to disease progression. While these studies made advancements in diagnostic and epidemiological understanding, they focused on two seasons or limited fruit-maturity stages, leaving the temporal progression of GRBV unresolved. Specifically, knowledge gaps persist regarding the mechanisms underlying seasonal titer fluctuations [17, 20] and the influence of viral genetic diversity on disease progression [7]. The current study expands prior efforts by integrating a multi-season, multi-stage analysis focused on a commercially important cultivar (Merlot). The experimental design offered greater temporal resolution than previous studies and new insights into the temporal dynamics of GRBV titer across both maturity stages and years since infection.

We attempted to elucidate the temporal patterns of GRBV and establish the relationship between virus copy number and maturity stages of grapevine development. Additionally, the study assessed the viral load in the grapevines across multiple seasons. To ensure reliable copy number assessment across different seasons, we used dPCR, which does not rely on standard curves. In addition, dPCR overcomes a few of the deficiencies in qPCR such as lower PCR efficiency and lower precision in measuring when the difference is less than two-fold between the samples and is less affected by inhibitors in the assay. While dPCR offers higher accuracy and sensitivity, it is not meant as a regular diagnostic tool due to its prohibitive cost and technical demands. However, the data from dPCR provide a crucial window for field-deployable tests, such as LAMP, and those based on biosensors, and reduce false negatives. A parallel study utilizing qPCR reported GRBV copy numbers for Merlot ranging from 2 to 5 on a log scale, from vineyards in New York and California [17]. However, it is not clear if the copy number was based on the mass of original tissue or on nucleic acid extracted. Conversely, our study, using dPCR, found viral loads ranging from 5 to 7 log units for the same variety in California.

Temporal dynamics of GRBV copy number highlight how maturity stages influence viral accumulation, with a notable increase observed in the 2021 season. These findings align with previous research, showing differences in viral load between the early and late growing seasons. However, the current study offers finer resolution than previous seasonal studies [17, 20], demonstrating not only that GRBV titer increases over time, but also specifically identifies véraison and harvest as the stages of highest viral accumulation. These patterns are consistent across multiple years, adding robustness to the maturity-stage-specific insight. Understanding viral load progression throughout the maturity stages offers valuable insights for vineyard management. The progression aligns with the grapevine’s physiological changes, where increased metabolic activity and sugar accumulation during fruit ripening may create a favorable environment for viral replication.

Further, to validate the year-to-year variance and GRBV progression across maturity stages, a more robust statistical approach was required to quantify the independent effects of maturity stage and year on virus accumulation. Therefore, a mixed-effect model was employed to determine these effects and statistically validate the observed temporal patterns in GRBV copy number.

The linear mixed model revealed a clear spatiotemporal pattern of viral progression, with a substantial increase in viral load during the harvest season. This progression likely contributes to the viral impact on grapevine physiology, especially during fruit ripening, when the vine is most vulnerable to disruptions in sugar accumulation and fruit quality. Moreover, GRBV has been shown to alter the berry cell wall composition, affecting the release of phenolic extractability during winemaking, a crucial factor in producing high-quality wines [24]. Previous studies indicated that GRBV infection led to the retention of phenolic compounds in the grape skins, which impacted the wine color and taste [3, 15]. Post-véraison showed no significance, while véraison exhibited a highly significant increase (*p* < 0.01) in viral copy number when compared to pre-véraison. Although viral load significantly increased from pre-véraison to véraison, the subsequent increase noticed at post-véraison was only marginally significant when compared to both pre-véraison and véraison, indicating a plateau or reduced rate of accumulation during this intermediate stage.

While maturity stages significantly impact viral load, multiple seasons or years since infection had no effect. According to the model, viral load increased slightly with each additional year, but this effect was not statistically significant, indicating that year-to-year variation does not strongly influence the viral load. This finding aligns with Flasco et al. [17], who also found no significant difference in viral load among grapevines infected with GRBV over seven years in a Cabernet Franc vineyard in California, in the 2022 growing season.

Compared to the previous report of Kahl et al. [20], the use of mixed effect modeling, an approach not common in GRBV studies, further validates the findings by isolating the influence of maturity stage from infection year, providing strong statistical validation for observed trends. The analysis reveals a clear spatiotemporal pattern in viral copy number progression with maturity stages emerging as the primary determinant of GRBV accumulation, while time since infection showed minimal influence. This likely reflects the close association between viral replication and grapevine developmental processes, supporting previous findings that GRBV can significantly reduce berry sugar content, delay ripening, and alter secondary metabolites, such as anthocyanins and volatile compounds, which are crucial for wine quality [3, 15].

The observation of reduced viral load at elevated temperatures was along established findings for GRBV and other plant viruses, suggesting a potential interaction between environmental stressors and viral progression. For instance, multiplication of tobacco mosaic virus (TMV) was significantly inhibited above 32 °C, with viral content declining after reaching a peak at temperatures above 28 °C [25]. Extreme temperatures, typically exceeding 30 °C, have been known to trigger plant defense responses, specifically RNA silencing, which could interfere with viral gene expression. Studies on tomato ringspot virus have shown that the elevated temperatures (27 °C vs. 21 °C) enhance the efficiency of the plant’s silencing machinery, leading to an earlier and more significant accumulation of viral small interfering RNAs and a reduction in viral protein accumulation [26]. This activation of RNA silencing can interfere with viral gene expression and replication. In the case of cucurbit leaf crumple virus, a bipartite begomovirus, this type of defense response, known as “recovery,” is directly associated with reduced viral DNA levels, increased viral genome methylation, and the accumulation of viral siRNAs, confirming gene silencing as a central mechanism [27].

The 2022 data highlighted the suppression of virus replication by heat stress introduces a key environmental factor that can account for interannual variability in the virus titer. Taken together, it is likely that GRBV may also undergo temperature-mediated suppression through similar silencing mechanisms, with environmental fluctuations in the vineyard directly shaping viral accumulation and symptom severity across seasons [15, 28]. However, while such mechanisms might have mitigated viral accumulation, they also underscore the vulnerability of grapevines to climate.

Our study provides useful information for GRBV infection dynamics to inform disease monitoring and diagnostic planning for vineyards. Extending previous regional research from British Columbia and Oregon, California vineyard data adds to the understanding of GRBV infection under warmer climatic conditions. Our research contributes to a climatically relevant interpretive framework for GRBD progression for premium wine production areas.

Our study provides practical insights using dPCR, rather than recommending it as a routine vineyard monitoring tool due to its cost and technical complexity. It shows, with high accuracy, that GRBV titers peak at véraison and harvest, guiding optimal sampling times for field diagnostic tests. Growers looking for affordable and reliable disease detection can conduct field-deployable tests on or after véraison and reduce the risk of false negatives. This is economically significant because missed detections can delay management by rogueing, leading to increased management costs and due to further spread by insect vectors. Years since infection did not affect the viral loads, stabilizing the diagnostic window and streamlining monitoring in vineyards with varied infection histories. Monitoring GRBV levels across growth stages may indicate how vines respond to long-term stress, aiding the interpretation of atypical symptoms. Improving detection thresholds based on dPCR data and integrating them into decision tools will enhance test reliability and support more strategic vineyard management.

## Supplementary Information

Below is the link to the electronic supplementary material.

Supplementary figure 1 (PNG 90.8 KB)

High Resolution Image (TIF 193 KB)

Supplementary Material 2 (PPTX 119 KB)

## Declarations

### Disclaimer

The use of trade, firm, or corporation names in this publication (or page) is for the information and convenience of the reader. Such use does not constitute an official endorsement or approval by the United States Department of Agriculture or the Agricultural Research Service of any product or service to the exclusion of others that may be suitable.
