# Supplementary figures and images for: Temporal dynamics of Grapevine red blotch virus accumulation in grapevine leaves is influenced by fruit maturity stages
Source: Arch Virol. 2026 May 20;171(6):183. doi: 10.1007/s00705-026-06634-0 (PMC13190339; doi:10.1007/s00705-026-06634-0)

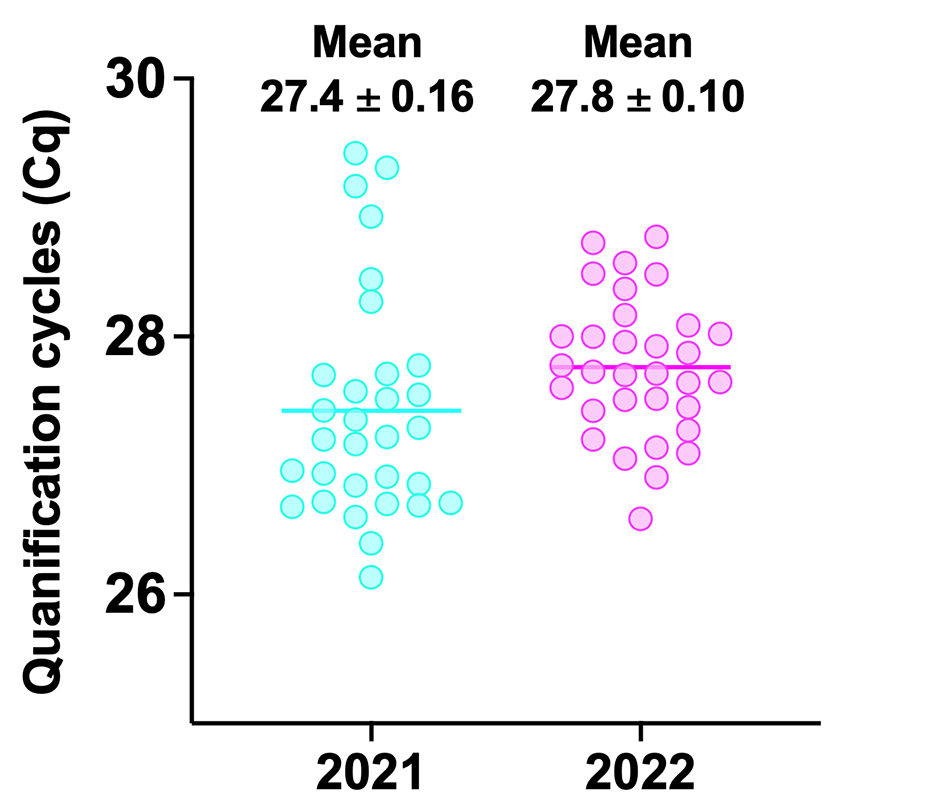

Supplement: Supplementary file 1 — (PNG 90.8 KB) [file 705_2026_6634_Fig6_ESM.png]

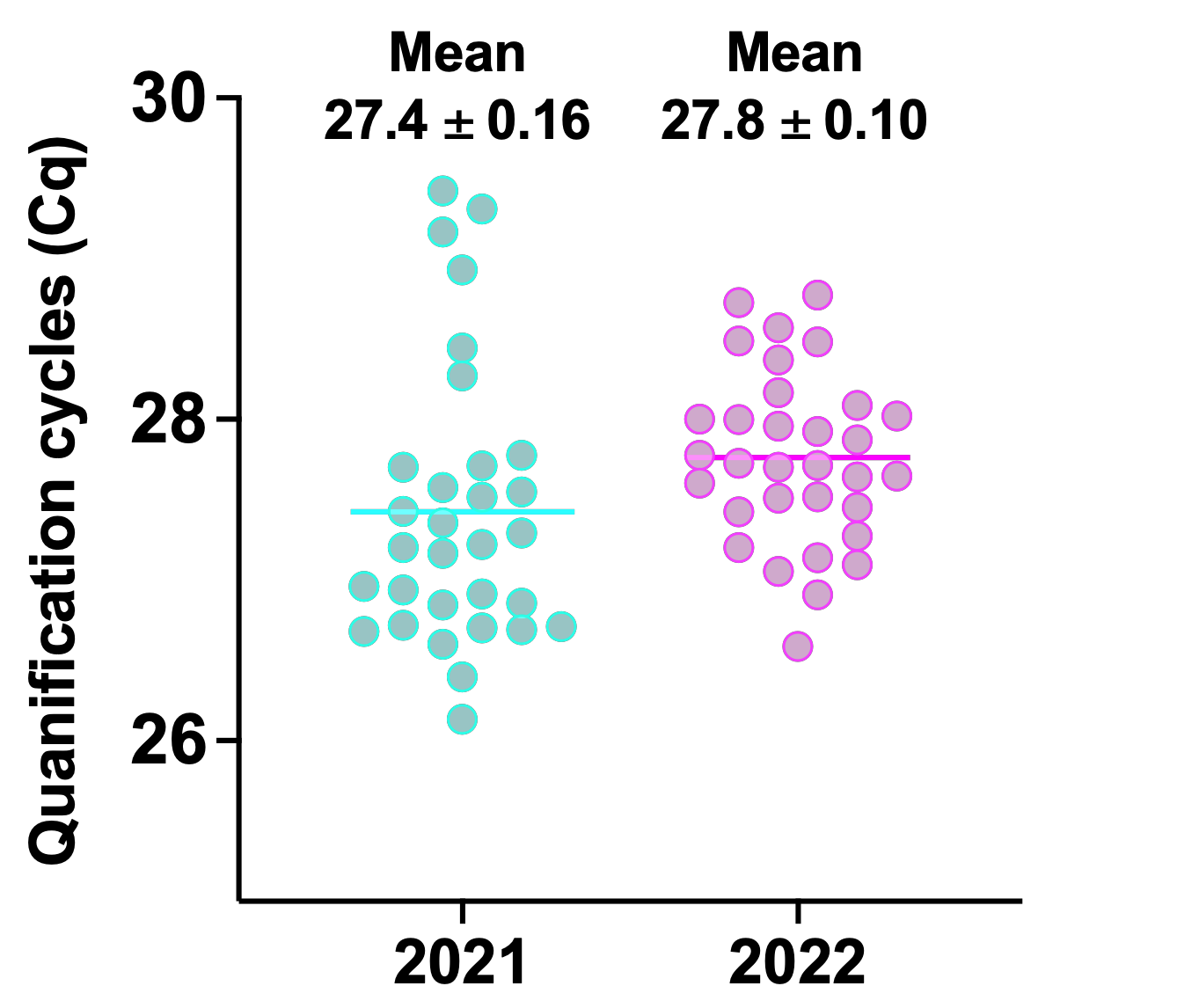

Supplement: Supplementary file 2 — High Resolution Image (TIF 193 KB) [file 705_2026_6634_MOESM1_ESM.tif]

## Slide 1
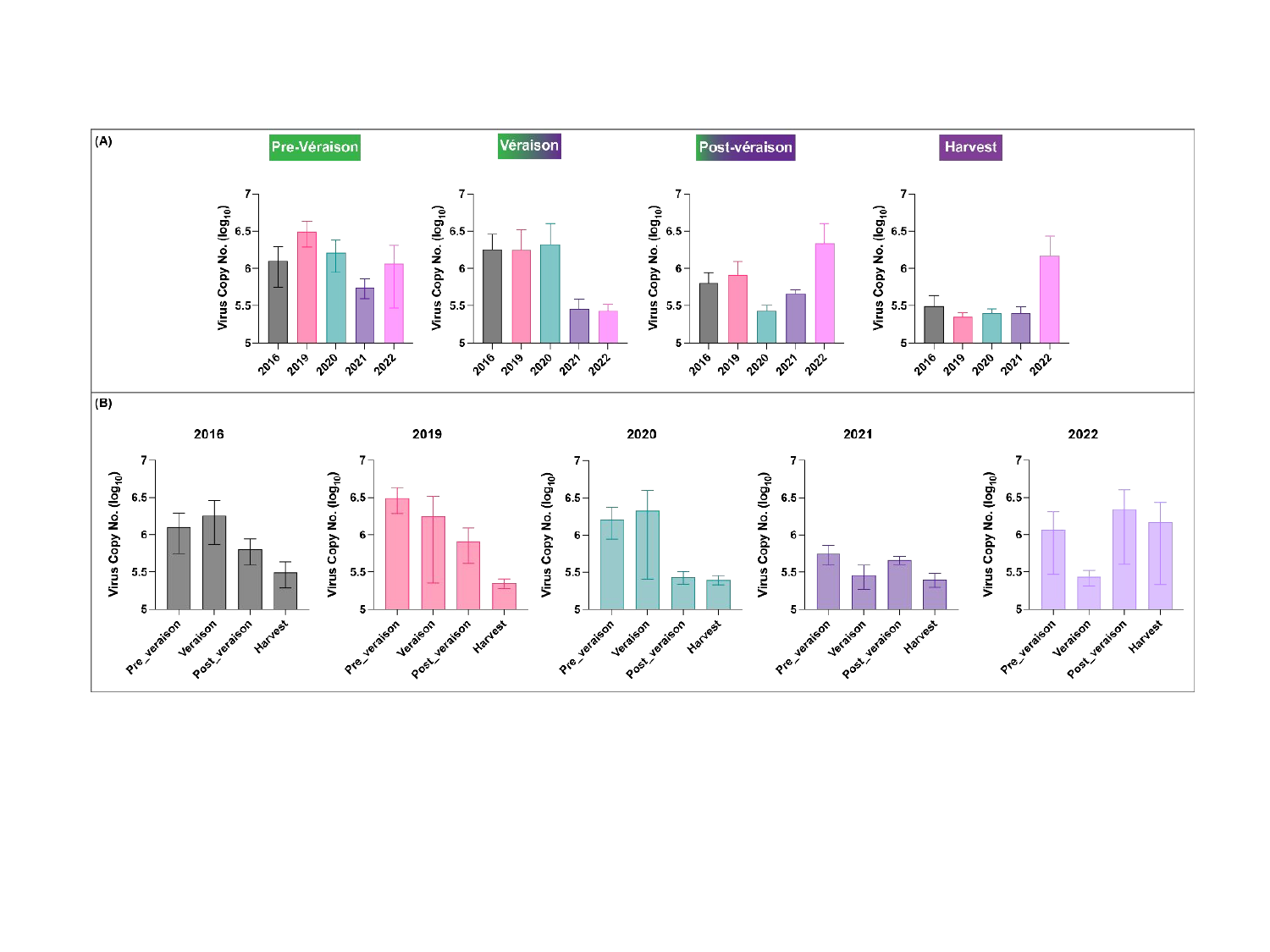

Supplement: Supplementary file 3 — Supplementary Material 2 (PPTX 119 KB) [file 705_2026_6634_MOESM2_ESM.pptx]
